# Supplementary material for: Can counterfactual explanations of AI systems’ predictions skew lay users’ causal intuitions about the world? If so, can we correct for that?
Source: Patterns (N Y). 2022 Dec 9;3(12):100635. doi: 10.1016/j.patter.2022.100635 (PMC9768678; doi:10.1016/j.patter.2022.100635)
Supplement: Document S1. Figures 4–8, Tables 1–4, and Appendices A and B [file mmc1.pdf]

Patterns, Volume *3*

Supplemental information

Can counterfactual explanations of AI systems' predictions skew lay users' causal intuitions about the world? If so, can we correct for that?

Marko Tešić and Ulrike Hahn

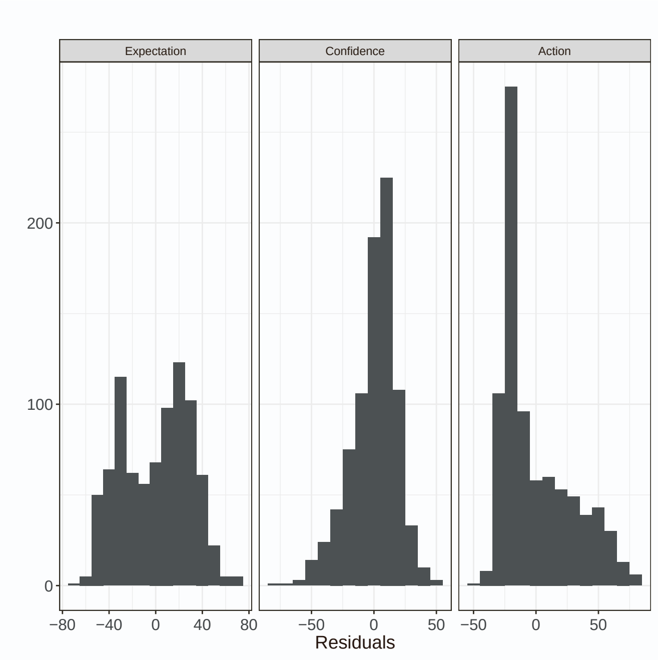

Figure 5: Histograms of the LMM residuals for all three dependent variables in Experiment 1.

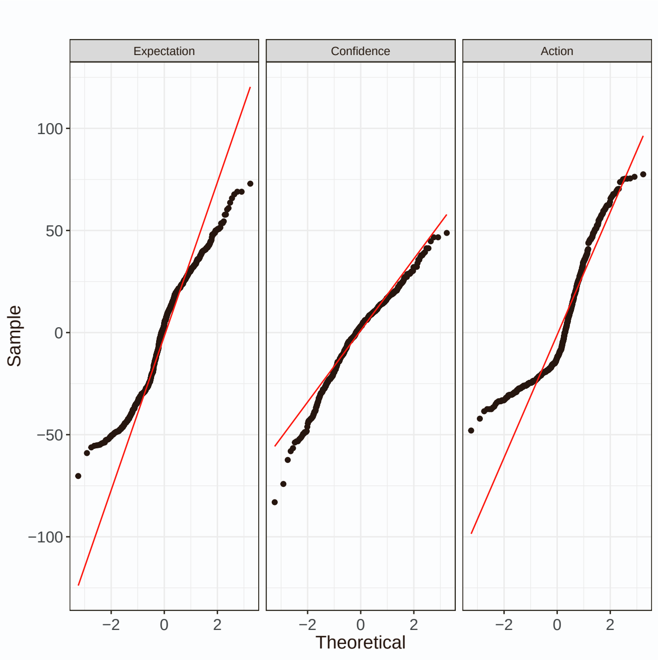

Figure 6: Quantile plots of the LMM residuals for all three dependent variables in Experiment 1.

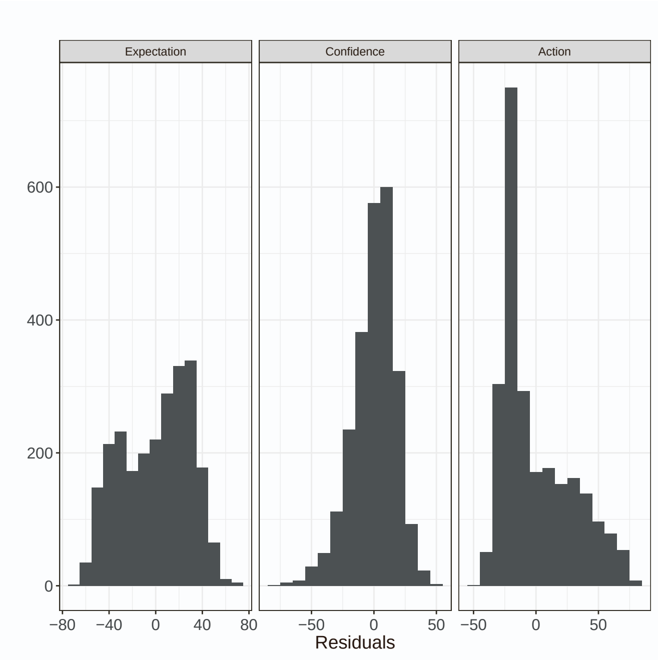

Figure 7: Histograms of the LMM residuals for all three dependent variables in Experiment 2.

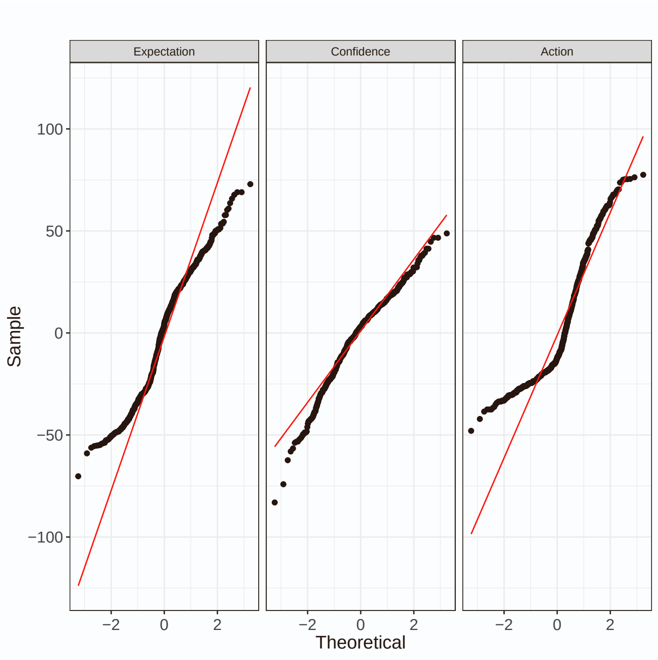

Figure 8: Quantile plots of the LMM residuals for all three dependent variables in Experiment 2.

Table 1: Experiment 1: Pairwise Wilcoxon Rank Sum tests p-values for all three dependent variables. All p-values were corrected for multiple comparisons using Benjamini and Hochberg's false discovery rate (FDR) procedure [Benjamini and Hochberg, 1995].

	Expectation		Confidence		Action	
	Control	AI Prediction	Control	AI Prediction	Control	AI Prediction
AI Prediction	.03		.02		.74	
AI Explanation	.002	.36	< .001	.12	< .001	< .001

Table 2: Experiment 2: Pairwise Wilcoxon Rank Sum tests p-values for dependent variable Expectation. All p-values were corrected for multiple comparisons using the false discovery rate method.

	Control & No Note	Control & Note	AI Prediction & No Note	AI Prediction & Note	AI Explanation & No Note
Control & Note	.33				
AI Prediction & No Note	.001	.02			
AI Prediction & Note	< .001	.004	.68		
AI Explanation & No Note	< .001	< .001	.31	.47	
AI Explanation & Note	< .001	.01	.65	.88	.59

Table 3: Experiment 2: Pairwise Wilcoxon Rank Sum tests p-values for dependent variable Confidence. All p-values were corrected for multiple comparisons using the false discovery rate method.

	Control & No Note	Control & Note	AI Prediction & No Note	AI Prediction & Note	AI Explanation & No Note
Control & Note	.08				
AI Prediction & No Note	.03	.77			
AI Prediction & Note	.001	.17	.28		
AI Explanation & No Note	.95	.08	.03	< .001	
AI Explanation & Note	.31	.008	.003	< .001	.28

Table 4: Experiment 2: Pairwise Wilcoxon Rank Sum tests p-values for dependent variable Action. All p-values were corrected for multiple comparisons using the false discovery rate method.

	Control & No Note	Control & Note	AI Prediction & No Note	AI Prediction & Note	AI Explanation & No Note
Control & Note	.63				
AI Prediction & No Note	< .001	< .001			
AI Prediction & Note	.001	.003	.39		
AI Explanation & No Note	< .001	< .001	.02	.001	
AI Explanation & Note	< .001	.002	.63	.63	.01

Appendix A: Pairwise comparisons

Table 1 shows post hoc pairwise comparison for each dependent variable in Experiment 1. Tables 2, 3, and 4 include all post hoc pairwise comparisons for each dependent variable in Experiment 2.

Appendix B: Linear mixed-effects models

B.1 Experiment 1

To estimate the effect of group on the three dependent variables, we initially built linear mixed-effects models (LMMs) using the "lme4" package in R.[S1] The only fixed effect was group (with three levels: control, AI prediction, AI explanation). The only random effect was the intercept for participants. There was no random slope from the participant as the design was fully between. No random intercept for scenarios was used as the number of scenarios was low (i.e., 9) and including the scenarios as a random intercept could have led to a reduced power of the experiment (see Judd et al.[S2] and Singmann and Kellen[S3]). Further, a random slope for scenarios was not included as this led to a singular fit model, implying that the variance of this random effect was (close to) zero.

After we fitted the LMM, we plotted the quantile plots of the residuals and the histograms of residuals. Figures 5 and 6 show that the residuals are non-normally distributed for all dependent variables. Consequently, we resorted to the non-parametric statistical analyses outlined in the main text.

B.2 Experiment 2

We build a similar LMM for experiment 2. The only difference was that instead of only one fixed effect, we now had two: condition (control, AI prediction, AI explanation) and correction (no note, note). The random effects structure was the same as in experiment 1. We again plotted the residuals and found that they were not normally distributed (see Figures 7 and 8). We then performed the same non-parametric analyses as in experiment 1.

Supporting references

S1. D. Bates, M. Mächler, Ben B., and S. Walker. (2014) Fitting linear mixed-effects models using lme4. arXiv https://doi.org/10.48550/arXiv.1406.5823.

S2. C.M. Judd, Westfall J., and D.A. Kenny. (2017) Experiments with more than one random factor: designs, analytic models, and statistical power. Annu. Rev. Psychol. 68, 601–625.

S3. H. Singmann and D. Kellen. (2019) An introduction to linear mixed modeling in experimental psychology. In *New Methods in Cognitive Psychology*, pp. 4–31. Psychology Press.
